# Multiple deficits in ADHD: executive dysfunction, delay aversion, reaction time variability, and emotional deficits

**DOI:** 10.1111/jcpp.12006

**Published:** 2012-10-15

**Authors:** Douglas Sjöwall, Linda Roth, Sofia Lindqvist, Lisa B Thorell

**Affiliations:** 1 Department of Clinical Neuroscience and Stockholm Brain Institute, Karolinska InstitutetStockholm, Sweden; 2 Department of Psychology, Uppsala UniversityUppsala, Sweden

**Keywords:** ADHD, executive function, emotion regulation, emotion recognition

## Abstract

**Background:**

The notion that ADHD constitutes a heterogeneous disorder is well accepted. However, this study contributes with new important knowledge by examining independent effects of a large range of neuropsychological deficits. In addition, the study investigated whether deficits in emotional functioning constitute a dissociable component of ADHD.

**Method:**

The study included children with ADHD (*n* = 102; 7–13 years) and a control sample individually matched with regard to age and gender. The administered tasks were designed to tap into three different neuropsychological domains: executive functions (i.e., working memory, inhibition, and shifting), delay aversion, and reaction time variability. Parent ratings of emotion regulation and a test of emotion recognition were also included.

**Results:**

Children with ADHD differed significantly from controls on all measures, except for delay aversion and recognition of disgust. No main effects of gender or interaction effects of gender and group were found. More importantly, executive functioning, reaction time variability, and emotional functioning all contributed independently to distinguishing between children with ADHD and controls.

**Conclusions:**

The current study supports the view of ADHD as a heterogeneous disorder related to multiple neuropsychological deficits. In addition, emotional functioning appears to be an area of importance for ADHD that needs to be incorporated into future theoretical models.

## Introduction

Recent theoretical models have emphasized the neuropsychological heterogeneity in Attention-deficit/Hyperactivity Disorder (ADHD; Castellanos, Sonuga-Barke, Milham, & Tannock, 2006; Nigg, Willcutt, Doyle, & Sonuga-Barke, 2005). Despite this, relatively few studies have investigated possible independent effects of different neuropsychological functions to address the question of whether different neuropsychological subtypes are present among children with ADHD. One exception is the dual-pathway model (Sonuga-Barke, 2002), in which ADHD is described as having two different pathways. The first pathway includes executive deficits such as working memory and inhibition, and the second pathway is defined as delay aversion (i.e., the tendency to choose a smaller immediate reward rather than wait for a larger delayed reward). Several studies (e.g., Dalen, Sonuga-Barke, Hall, & Remington, 2004; Solanto et al., 2001; Sonuga-Barke, Dalen, & Remington, 2003) have found that ADHD is significantly related to delay aversion and that this deficit is independent of deficits in inhibitory control. However, other studies have failed to find a relation between delay aversion and ADHD (e.g., Karalunas & Huang-Pollock, 2011; Solanto et al., 2007). The inconsistencies in previous findings may be related to age, as it has been argued that effect sizes for delay aversion are largest in younger samples (e.g., Karalunas & Huang-Pollock, 2011). In addition, it has been shown that a relatively large number of children with ADHD are not deficient with regard to either executive functions or delay aversion (Nigg et al., 2005), making it necessary to include more factors if we are to fully understand the deficits associated with ADHD.

A third factor that has been shown to be strongly related to ADHD is reaction time (RT) variability (e.g., Castellanos et al., 2005 for a review). This is believed to arise from an inability to mobilize an appropriate amount of energy in relation to what the task or the situation requires (Sergeant, 2005). However, the exact nature of high RT variability in ADHD is still uncertain (cf. Castellanos et al., 2005; Sonuga-Barke, Wiersema, van der Meere, & Roeyers, 2010). To our knowledge, only two previous studies have investigated whether RT variability is related to ADHD independent of both executive functioning and delay aversion. The first study found independent effects of working memory, RT variability, and delay aversion (Kuntsi, Oosterlaan, & Stevenson, 2001), whereas the second study found independent effects of RT variability and inhibition only (Wåhlstedt, Thorell, & Bohlin, 2009).

In addition to executive functioning, delay aversion, and RT variability, the importance of considering the role of emotional functioning in ADHD has been acknowledged (e.g., Martel, 2009; Nigg, 2006). Compared to executive functioning, relatively little research has examined emotional functioning in clinical ADHD samples, although a few studies have shown that ADHD is related to deficiencies in both emotion regulation (e.g., Maedgen & Carlson, 2000; Walcott & Landau, 2004) and emotion recognition (e.g., Kats-Gold, Besser, & Priel, 2007; Sinzig, Morsch, & Lehmkuhl, 2008; Yuill & Lyon, 2007). Even fewer clinical studies have examined to what extent emotional impairments are independent of neuropsychological impairments in ADHD. Nonclinical studies have suggested that executive and emotional control develop in concert during the preschool period (e.g., Carlson & Wang, 2007), and Barkley (1997) proposed that inhibitory control is the primary deficit in ADHD, which leads to secondary problems with emotion regulation. On the other hand, there are some clinical data available suggesting that neuropsychological and emotional processes are independent in ADHD (Berlin, Bohlin, Nyberg, & Janols, 2004; Blaskey, Harris, & Nigg, 2007), but a rather limited number of neuropsychological functions were included in these studies. Finally, it should be noted that previous studies have often failed to control for comorbid conduct problems, even though it has been suggested that poor emotion regulation may be primarily evident in the subgroup of children with ADHD who are highly aggressive (Melnick & Hinshaw, 2000). In addition, emotion regulation deficits, especially poor regulation of fear, have been shown to be related to internalizing problems (e.g., Rydell, Berlin, & Bohlin, 2003). Thus, it is important to also take comorbid problems of the internalizing kind into consideration.

### Aim of the present study

The aim of this study was to examine possible independent effects of a range of different neuropsychological deficits in ADHD, and to investigate whether deficits in emotional functioning constitute a dissociable component of ADHD. As most previous studies have failed to investigate gender differences, we also aimed to explore this issue. Multiple analytic approaches were used. First, simple group differences were studied and a logistic regression analysis was thereafter used to investigate independent effects of different neuropsychological and emotional functions. Next, we followed the suggestion made by, for example, Nigg et al. (2005), who emphasized the need for more person-centered (rather than variable-centered) research in ADHD samples. This was accomplished by categorizing children as “impaired” versus “unimpaired” with regard to neuropsychological and emotional functioning and illustrating the overlap using Venn diagrams (see Statistical Analyses for a more detailed description).

On the basis of the results from the previous studies described above, we made the following hypotheses. First, we hypothesized that children diagnosed with ADHD would perform more poorly compared to controls on tests measuring executive functioning (i.e., working memory, inhibition, and shifting), RT variability as well as delay aversion. Moreover, we hypothesized that these neuropsychological deficits would not fully overlap, but display independent contributions when studied simultaneously. Finally, we hypothesized that children with ADHD would perform more poorly than controls with regard to emotion regulation and emotion recognition. Due to the scarcity of previous studies examining the link between neuropsychological and emotional functioning, no a priori hypotheses were made with regard to this issue. In line with a recent review (Rucklidge, 2010), we hypothesized that no gender differences would be seen in executive functioning. Due to the very limited amount of clinical data available on gender differences in delay aversion, RT variability, and emotional functioning, no a priori hypotheses were made.

## Methods

### Participants

This study included 102 children (56 girls) aged 7–13 years and diagnosed with ADHD and a control group of 102 children individually matched to the clinical group with regard to gender and age (±6 months). The ADHD sample included clinically referred children who were recruited from Stockholm ADHD center and child and adolescent psychiatric clinics. As one aim was to study gender differences, girls were oversampled. All children had been formally diagnosed with ADHD by a psychiatrist, and the children’s diagnostic status was also confirmed at the time of the study using the ADHD Rating Scale IV (DuPaul, Power, Anastopoulos, & Reid, 1998), which includes the 18 symptoms of ADHD as presented in DSM-IV (American Psychiatric Association, 1994). In line with DSM-IV criteria, we also used the impact supplement from the Strength and Difficulties Questionnaire (SDQ; Goodman, 1997) to confirm that the symptoms had been present before age 7, for at least 6 months, and that impairment was found in multiple settings. Based on teacher and parent ratings, 71 children (70%) met the criteria for the combined subtype, four children (4%) met the criteria for the hyperactive/impulsive subtype, and 27 children (26%) met the criteria for the inattentive subtype. Comorbid diagnoses included oppositional defiant disorder or conduct disorder (46%), generalized anxiety disorder/anxiety NOS (7%), obsessive compulsive disorder (1%), and Tourette syndrome (4%). Children with an IQ < 70 were excluded from the study. All children receiving psychopharmacological treatment for ADHD were asked to withdraw medication 24 hr prior to testing. For two participants, the child’s parents did not consent to take the child off medication. In addition, eight parents gave their consent, but forgot this despite having received a reminder. However, excluding the 10 children who were on medication at the time of the testing did not result in any significant changes in the results.

The control group was recruited by contacting schools in the Stockholm-Uppsala area in Sweden. Schools were chosen so that families of different socioeconomic status would be represented. The exclusion criteria for the control group were: (a) severe psychiatric or somatic problems as reported by parents and (b) scores above the 75th percentile on either the inattention or the hyperactivity subscale on the ADHD Rating Scale IV (DuPaul et al., 1998) as measured by teachers or parents. Controls and children with ADHD did not differ significantly with regard to parental education, both χ^2^ ≤ .61, ns, parental age, both ts ≤ 1.64, ns, number of siblings, *t* = .47, ns, nonverbal intelligence, *t* = 1.37, ns, and birth country of the parents or the child, all χ^2^ ≤ 2.49, ns.

### Procedure and measures

The study was approved by the ethics committee at the Karolinska Institute, Stockholm, Sweden.

#### Neuropsychological assessment

The tests were chosen based on previous research identifying three major aspects of executive functioning: working memory, inhibition, and shifting (e.g., Miyake, Friedman, Emerson, Witzki, & Howerter, 2000; Willcutt et al., 2001). All measures were standardized and some measures were reversed so that high values always indicated poor performance.

Working memory was measured using three tasks: one spatial and two verbal. Spatial working memory was measured using the ‘Find the phone task,’ which is similar in design to the spatial working memory task included in the Cambridge Neuropsychological Test Automated Battery (CANTAB; Owens, Downes, Sahakian, Polkey, & Robbins, 1990). In our version, telephones were shown on the computer screen and the task was to remember which telephone that had already rung to avoid selecting that phone several times. The number of times the children returned to a phone that had already rung was used as a measure of working memory deficits. The Children’s Size-Ordering Task (McInerney, Hrabok, & Kerns, 2005) measured verbal working memory. The test administrator read a list of well-known nouns (e.g., pencil, mountain, train) to the participant, and the task was to remember the words and then organize them in order of size of the named object (from small to large). The number of word pairs that the child produced in the correct order was used to measure working memory. Verbal working memory was also measured using the total score for the backward condition of the digit span subtest (Wechsler, 1991). Individual scores were standardized and aggregated into one composite score (rs = .34–.50, *p* < .001).

Inhibition was measured using two tasks. The first task was the go/no-go task developed by Berlin and Bohlin (2002). Inhibition was measured as commission errors (i.e., pushing the button when a no-go target was displayed). The second task was a Navon-like task used by, for example, Miyake et al. (2000). A circle consisting of small squares, or the opposite, a square consisting of small circles, was displayed on the computer screen. In one session, the participants were asked to respond to the local stimuli (e.g., the small squares making up the circle) and in the other session they were asked to respond to the global stimuli (e.g., the circle made up by the squares). These two sessions were randomized and the child responded to the stimuli by pressing a key to the left (circle) or right (square) on the computer keyboard. In each session, 20 objects (10 squares and 10 circles) were shown. The objects were displayed for 500 ms and the participant had 3,500 ms to give an answer. The score used was number of errors during each session. Individual scores were standardized and aggregated into one composite score (rs = .26–.33, *p* < .001).

Shifting was measured using the Navon-like task (see description above). A third trial was performed in which participants had to shift between responding to the local or the global stimuli. A square and a circle in the lower corners of the computer screen indicated what stimulus to respond to (local trials = small circle/square, global trials = large circle/square). In line with recommendations by Davidson, Amso, Anderson, and Diamond (2006), number of errors was used to measure shifting. Two children in the ADHD group had missing data due to failure to understand the instructions.

Delay aversion was measured using the Choice Delay Task (Sonuga-Barke, Taylor, Sembi, & Smith, 1992). Participants chose between an immediate small reward (2 s for one point) and a delayed large reward (30 s for 2 points). Delay aversion was measured as the number of times participants chose the small, immediate reward during the final 10 trials.

Reaction time variability was measured as the standard deviation of participants’ reaction time for correct answers on the two nonshifting trials in the Navon-like task and correct answers on the go/no-go task (see descriptions above). Individual scores were standardized and aggregated into one composite score (*r* = .36–.65, *p* < .001).

#### Emotional functioning

Emotion regulation was measured through parental ratings (70% mothers, 9% fathers, and 21% both) using the Emotion Questionnaire developed by Rydell et al. (2003). It includes statements related to regulation of anger, fear, sadness, and happiness/exuberance. For each emotion, one general question is asked (e.g., If sad, my child has trouble calming down by him-/herself) and two questions regarding regulation in specific situations (e.g., if my child has fallen and hurt him-/herself, my child has trouble calming down by him-/herself). Ratings are made on a scale ranging from 1 (do not agree at all) to 5 (fully agree), with higher values indicating greater problems with emotion regulation. This instrument has been shown to have high test-retest reliability and high construct validity (Rydell et al., 2003). Six ADHD children had missing data as their parents did not consent to completing the rating scale.

Emotion recognition was measured using facial images selected from the NimStim Set of Facial Expressions (672 images; http://www.macbrain.org/resources.htm), which consists of naturally posed photographs (e.g., with hair, make-up) of 43 professional actors (25 male; 21 to 30-years-old). In this study, the children were shown 36 faces displaying six different emotions: anger, fear, sadness, happiness, surprise, and disgust. One ADHD child had missing data. The score used was number of correct responses (maximum score = 6).

#### Control variables

Conduct problems and internalizing problem behaviors were measured using the mean of parent and teacher ratings on the SDQ (Goodman, 1997). Intelligence (IQ) was measured using the block design subtest from the WISC-III (Wechsler, 1991), which has been shown to correlate highly with full-scale IQ (*r* = .93; Groth-Marnat, 1997). The results are first reported without the control for these variables and the analyses were thereafter rerun to examine whether the pattern of results would hold after control for comorbid problems and intelligence.

### Statistical analyses

First, correlations were used to study relations within the neuropsychological variables and between the neuropsychological and the emotional variables. Second, two-way ANCOVAs were conducted with group and gender as fixed factors and the measures of neuropsychological and emotional functioning as dependent variables. Effect sizes were calculated using η^2^ (Cohen, 1988). To investigate independent effects, a logistic regression analysis was performed with group (ADHD vs. control) as the dependent variable. The measures of neuropsychological functioning were entered in the first step and measures of emotional functioning in the second step. From the results of the logistic regression analysis, measures of sensitivity (i.e., the percentage of children with ADHD who were correctly classified) and specificity (i.e., the percentage of children in the control group who were correctly classified) were also calculated.

Finally, a categorical characterization was made in which participants were classified as ‘impaired’ or ‘unimpaired.’ In line with several other studies (e.g., Nigg et al., 2005; Sonuga-Barke, Bitsakou, & Thompson, 2010; Wåhlstedt et al., 2009), the 90th percentile of the control group was used as the cutoff for what was considered impaired. The scores were age-adjusted using standard regression procedures. Group and gender differences in these categorical measures were examined using chi-square analyses.

Age was used as a covariate in the correlations and in the ANCOVAs, as (a) all neuropsychological variables except delay aversion were significantly related to age, rs > .24, *p* < .001, and (b) boys were significantly older than girls, *t* = 3.16, *p* < .001. For the remaining analyses, age was not controlled for, as the groups (ADHD and controls) were matched with regard to this variable. However, to address the question of whether group differences were equally large across the age span studied, we divided the sample into three equally large age groups and investigated interactions of group (ADHD vs. controls) and age group. As the pattern of results was found to be similar for all three age groups, these findings are not further described in the results section below. Gender was used as a separate factor in the ANCOVAs, as one aim of the study was to examine main effects of gender and interaction effects of group and gender. For the other control variables, the results were first presented without control for intelligence, internalizing problems, and conduct problems. However, the analyses were thereafter rerun including these covariates and changes in the results are reported. Univariate outliers were defined as children within each group (ADHD vs. controls) with a value above or below 3 *SD*. Outliers were handled by replacing the extreme value with the value at 3 *SD*. This procedure eliminated the impact of outliers and allowed us to retain all children in the analyses.

## Results

Correlation analyses showed that the three measures of executive functioning were significantly interrelated, and RT variability was related to all three measures of executive functioning (see Table 1). Furthermore, delay aversion was significantly related to working memory and inhibition. Executive functioning and RT variability showed significant relations to almost all measures of emotional functioning. Delay aversion, on the other hand, was only significantly related to recognition of sadness.

**Table 1:** Correlations within and between the neuropsychological variables and the variables measuring emotional functioning using age and gender as covariates. Percentage of children with neuropsychological or emotional impairment based on the 90th percentile of the controls

						Impairment (%)	
						
	Inhibition	Working memory	Shifting	RT variability	Delay aversion	Boys	Girls
Neuropsychological deficits							
Inhibition	-					24	25
Working memory	.40***	-				22	25
Shifting	.50***	.42***	-			28	30
Reaction time variability	.44***	.40***	.33***	-		50	57
Delay aversion	.14*	.28***	.10	.08	-	17	11
Emotion recognition							
Anger	−.28***	−.28***	−.27***	−.33***	−.03	33	49
Sadness	−.18**	−.20**	−.34***	−.18**	−.18*	24	22
Fear	−.22**	−.24***	−.19**	−.26***	−.10	26	13
Happiness	−.12	−.14	−.27***	−.23***	.04	22	22
Disgust	−.09	−.20**	−.12	−.09	.06	13	9
Surprise	−.22**	−.21**	−.21**	−.15*	−.07	24	18
Emotion regulation deficits							
Anger	.21**	.22**	.21*	.43***	.07	61	65
Sadness	.16*	.17*	.15*	.34***	.03	61	50
Scared	.27***	.17*	.25***	.34***	.03	34	42
Happiness	.17*	.17*	.16*	.30***	−.01	52	48

* *p* < .05

** *p* < .01

*** *p* < .001.

When studying group differences, the children with ADHD performed more poorly than controls with regard to all neuropsychological functions except delay aversion (see Table 2). Moreover, group differences were seen for all measures of emotional functioning, except for recognition of disgust. Effect sizes for the significant group differences were medium to large (η^2^ = .09–.27) for the neuropsychological measures, large (η^2^ = .22–.44) for emotion regulation, and medium to large (η^2^ = .05–.15) for emotion recognition. No main effects of gender and no significant interactions of group and gender were found. All group differences remained significant when controlling for multiple comparisons (i.e., Bonferroni). In addition, all group differences remained significant when controlling for either IQ, conduct problems, or internalizing problems, except for recognition of sadness, which did not remain significant when controlling for internalizing problems.

**Table 2:** Means, standard deviations, and results of the two-way ANCOVA (controlling for age)

	ADHD group		Control group		two-way ANCOVAs		
			
	Boys	Girls	Boys	Girls	Group	Sex	Group × Sex
							
	*M* (*SD*)	*M* (*SD*)	*M* (*SD*)	*M* (*SD*)	F	F	F
Neuropsychological deficits^a^							
Inhibition	0.41 (1.12)	0.27 (1.00)	−0.33 (0.70)	−0.36 (0.72)	31.60***	2.87	0.18
Working memory	0.25 (0.93)	0.36 (0.99)	−0.29 (1.08)	−0.28 (0.83)	23.36***	0.96	0.22
Shifting	0.32 (1.01)	0.27 (1.02)	−0.28 (0.82)	−0.29 (0.86)	20.54***	1.19	0.01
RT variability	0.32 (1.04)	0.71 (1.04)	−0.56 (0.62)	−0.46 (0.57)	81.81***	1.17	1.87
Delay aversion	0.09 (1.13)	0.07 (1.03)	−0.23 (1.04)	0.03 (0.81)	1.61	0.54	0.98
Emotion recognition^b^							
Anger	4.59 (1.37)	4.42 (1.38)	5.48 (0.81)	5.43 (0.83)	34.92***	0.43	0.15
Sadness	3.22 (1.01)	3.42 (1.01)	3.60 (0.97)	3.89 (0.68)	10.85***	3.42	0.13
Fear	2.65 (1.48)	2.87 (1.17)	3.59 (1.42)	3.68 (1.45)	19.83***	0.77	0.11
Happiness	5.20 (0.92)	5.20 (0.98)	5.65 (0.65)	5.70 (0.54)	17.68***	0.10	0.04
Disgust	4.13 (1.54)	4.25 (1.36)	4.36 (1.41)	4.36 (1.24)	0.70	0.11	0.10
Surprise	3.52 (1.39)	3.85 (1.30)	4.30 (1.38)	4.27 (1.05)	11.50***	2.81	1.04
Emotion regulation deficits^c^							
Anger	3.42 (1.14)	3.44 (1.05)	1.83 (0.77)	1.81 (0.68)	151.20***	0.03	0.02
Sadness	3.26 (1.06)	3.32 (0.95)	2.12 (1.00)	2.01 (0.88)	79.26***	0.45	0.39
Scared	2.90 (1.22)	3.19 (1.11)	1.86 (0.85)	2.04 (0.86)	56.81***	1.24	0.15
Happiness	3.37 (1.01)	3.36 (1.10)	2.01 (0.98)	2.05 (0.81)	90.23***	0.00	0.03

a Standardized measures.

b Measured on a scale from 1 to 5.

c Measured as correct responses (maximum = 6).

*** *p* < .001.

Next, a logistic regression analysis was performed, including the variables for which a significant group difference had been found. In the first step, there was a significant effect of RT variability, Wald = 27.09, *p* < .001, and a near significant effect of inhibition, Wald = 3.80, *p* = .05, but no significant effects of shifting and working memory, Walds < 1.81, ps > .18. In the second step, there were significant effects of anger recognition, Wald = 6.08, *p* < .05, regulation of anger, Wald = 19.60, *p* < .001, and regulation of happiness, Wald = 4.49, *p* < .05. The model successfully predicted 64.9% of the ADHD cases (i.e., sensitivity) and 84.3% of the controls (i.e., specificity) after the first step and 91.5% of the ADHD cases and 87.3% of the controls after the second step.

Thereafter, categorical analyses were conducted by defining impairment as performing poorer than the 90th percentile of the children in the control group. RT variability was the most common neuropsychological impairment and anger regulation the most common emotional impairment (see Table 1). Chi-square analyses showed that the group difference for recognition of fear was only marginally significant, χ^2^ < 3.36, *p* = .07. However, for the remaining variables, the results were identical to the ANCOVAs, with ‘impairment’ being significantly more common among children with ADHD compared to controls for all variables except delay aversion and recognition of disgust; all significant χ^2^ > 18.97, *p* < .001. Also in line with the ANCOVAs, no significant gender differences were found (all χ^2^ < 2.93) and the following analyses will therefore not be presented separately for boys and girls.

**Figure 1:**
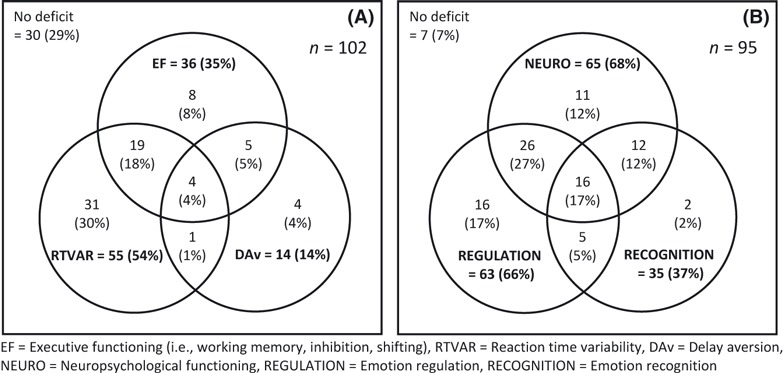
Proportion of ADHD cases with neuropsychological impairments (A) or impairments in neuropsychological and emotional functioning (B)

Figure 1(A) presents a Venn diagram showing the overlap between different types of neuropsychological impairment. To simplify the presentation of this categorical data, a mean value was computed for executive functioning. The results showed that 71% of the children with ADHD were shown to have at least one type of neuropsychological impairment: executive functioning (35%), RT variability (54%), and delay aversion (14%). Only four children were shown to have impairments in delay aversion that did not overlap with impairment in the other two domains. Among the remaining 68 children with impairment in either RT variability or executive functioning, there was a substantial overlap (23 children having deficits in both these functions), but also subgroups with impairment in either executive functioning (13 children) or RT variability (32 children). Among the controls, 26% had at least one neuropsychological deficit.

Next, we investigated the overlap between impairments in neuropsychological and emotional functioning (see Figure 1B). To be classified as impaired in neuropsychological functioning, the children had to be impaired with regard to at least one neuropsychological function (i.e., executive functioning, delay aversion or RT variability). For emotion recognition and emotion regulation, we computed two mean values. As with the other measures, impairment was thereafter defined as performing poorer than the 90th percentile of the children in the control group. The results showed that only 7% of the children with ADHD, but 61% of the controls, were not impaired in any domain. Among the children with ADHD, 12% were impaired only in neuropsychological functioning, 24% were impaired only in emotional functioning, and 57% had impairments in both domains. Only two children had impairment in emotion recognition that did not overlap with impairment in either neuropsychological functioning or emotion regulation. Among the children in the control group, 21% were impaired only in neuropsychological functioning, 13% were impaired only in emotional functioning, and only 6% had impairments in both domains.

## Discussion

In line with current theoretical models of heterogeneity in ADHD (e.g., Castellanos et al., 2006; Nigg et al., 2005), this study aimed to explore independent effects of different neuropsychological functions in ADHD and to investigate whether deficits in emotional functioning might constitute a dissociable component of ADHD. The results showed that the children with ADHD performed more poorly than controls on all variables except delay aversion and recognition of disgust. For the neuropsychological variables, independent effects were only seen for RT variability and inhibition. Furthermore, emotion regulation and emotion recognition showed independent effects beyond the influence of neuropsychological deficits and improved our ability to successfully distinguish between ADHD cases and controls. No significant gender differences were found.

### Neuropsychological deficits

In line with a large number of previous studies, this study found that children with ADHD performed more poorly than their typically developing peers on measures of executive functioning (for reviews, see Barkley, 1997; Nigg, 2006) and RT variability (e.g., Castellanos et al., 2005). However, when classifying children as impaired or unimpaired, only about one third of the children with ADHD were impaired with regard to executive functioning and about half were impaired with regard to RT variability. This finding is in line with previous studies (Nigg et al., 2005; Wåhlstedt et al., 2009) and supports the view that any reasonable cutoff will classify many children with ADHD as unimpaired or classify an inordinate number of control children as impaired (cf. Nigg et al., 2005). This pattern of results was found even though this study examined a larger number of neuropsychological functions compared to most previous studies. We conclude that executive functioning deficits and high RT variability are important characteristics of some, but not all, children with ADHD. When studying specific functions, RT variability and inhibition appeared to be of greatest importance. This conclusion, which corroborates the findings of Wåhlstedt et al. (2009), is based on the result showing that only RT variability and inhibition independently predicted group status in the logistic regression analysis.

In contrast with the dual-pathway model (Sonuga-Barke, 2002), no significant group differences were found for delay aversion and only 14% of the children with ADHD were defined as impaired in the categorical analyses. As mentioned in the introduction, previous research in this area has not been consistent. Some studies have shown significant group differences (e.g., Dalen et al., 2004; Solanto et al., 2001), whereas others have failed to do so (e.g., Karalunas & Huang-Pollock, 2011; Solanto et al., 2007). One possible explanation for our lack of group differences for delay aversion is the age of our sample, as it has been suggested effect sizes for delay aversion are largest in younger samples (Karalunas & Huang-Pollock, 2011). Future studies need to examine whether the tasks commonly used to study delay aversion are less appropriate for older children. Temporal discounting tasks may be a better option for older children and adolescents, although it should be noted that previous ADHD studies are inconsistent also with regard to this task paradigm (e.g., Barkley, Edwards, Laneri, Fletcher, & Metevia, 2001; Scheres et al., 2006).

### Emotional functioning

The results of this study showed that children with ADHD differed significantly from controls on most measures of emotional functioning, which is in line with previous studies demonstrating such differences with regard to both emotion regulation (e.g., Berlin et al., 2004; Maedgen & Carlson, 2000; Melnick & Hinshaw, 2000; Walcott & Landau, 2004) and emotion recognition (e.g., Kats-Gold et al., 2007; Sinzig et al., 2008; Yuill & Lyon, 2007). However, more importantly, this study contributed several new important findings. First, the study controlled for both conduct problems and internalizing problems and was able to show that emotional deficits are not related to ADHD solely because these children have higher levels of these comorbid problems.

Second, this study is the first ADHD study to examine emotional functioning in relation to a large number of neuropsychological functions, spanning across the most central models of ADHD. The results showed that three measures of emotional functioning (anger regulation, anger recognition, and regulation of happiness/exuberance) contributed significantly to discriminating between children with ADHD and controls, over and above the influence of neuropsychological functioning. In addition, the categorical analyses showed that a substantial subgroup of children with ADHD (24%) was affected with regard to emotional, but not neuropsychological, functioning.

A third important contribution of this study was the result showing that regulation of both positive and negative emotions contributed independently to the prediction of ADHD status. The effect of anger regulation is not surprising considering that comorbid aggression is common in children with ADHD. With regard to happiness/exuberance, our findings are in line with previous studies using the same rating scale, which have found a relation between regulation of happiness/exuberance and externalizing behavior problems in a nonclinical sample using either parent ratings (Rydell et al., 2003) or self-ratings (Rydell, Thorell, & Bohlin, 2007). More importantly, this finding supports the notion that ADHD is associated with disruptions in positive emotions or approach systems, as suggested by, for example, Martel and Nigg (Martel, 2009; Martel & Nigg, 2006).

Finally, it should be noted that when reanalyzing data from several studies, Nigg et al. (2005) showed that about 60%–70% of their sample was categorized as impaired when measures of inhibition and delay aversion were included. Neither this study nor previous studies (Sonuga-Barke, Bitsakou, et al., 2010; Wåhlstedt et al., 2009) have been able to improve this rate substantially by including a broader range of neuropsychological tasks. When we included emotional functioning in this study, more than 90% of the children in the ADHD group were shown to be impaired. It should be emphasized, however, that our cutoff (i.e., 90th percentile of the control group for each domain) was relatively liberal and 39% of the controls were also shown to be impaired in at least one domain. The primary aim of this study was to investigate independent effects of different neuropsychological deficits, and to clarify to what extent deficits in emotional functioning constitute a dissociable component of ADHD. Thus, we have contributed to the ADHD literature by showing that emotion regulation deficits are of central importance to ADHD and that these deficits only partly overlap with deficits in neuropsychological functions such as inhibition, working memory, and RT variability. However, it is for future research to collect normative data so that the best cutoff for different measures (i.e., showing the best balance between positive and negative predictive power) can be determined.

### Gender

This study had the advantage of including ADHD children and controls of both genders so that boys and girls with ADHD could be compared with one another as well as with same-sex controls. No main effects of gender and no interaction effects of group and gender were found. This is in line with a review by Rucklidge (2010), who concluded that boys and girls with ADHD have a very similar profile of executive dysfunction. Interestingly, this study demonstrated that this lack of gender differences also holds for RT variability, delay aversion, and emotional functioning.

## Limitations

The findings presented here must be addressed within the limitations of the study. First, no standardized clinical interview was used. However, all children were diagnosed by a licensed child psychiatrist and all diagnoses were confirmed using teacher and parent ratings on a standardized rating instrument. In addition, the children’s symptoms were reported to cause significant impairment in several settings and both the duration and age-of-onset criteria were met. A second limitation was that the delay aversion was assessed using only one task, which may have contributed to the low rates of impairment. Future studies should preferably use several tasks to measure both delay aversion and related constructs so as to obtain a clearer picture of motivationally based deficits in ADHD. Third, emotion regulation was assessed using ratings, and it is possible that ratings reflect a generalized parental view of the child as problematic rather than the specific ability to self-regulate. Speaking against this interpretation is the fact that our rating measure has been validated in relation to children’s self-reports (Rydell et al., 2007) and only moderate relations have been found between emotion regulation and emotional intensity (Rydell et al., 2003).

## Future directions and conclusions

With regard to future directions, the role of neuropsychological functions in providing valid information for diagnosis should be discussed. Looking at the discriminatory ability in this study, we can conclude that the classification rate was too low to regard deficits in neuropsychological functions as a viable replacement for the behavioral symptoms assessed in the current version of the DSM. Adding measures of emotional functioning improved the classification rate substantially. However, it is for future research to examine the role of emotional deficits in discriminating between different clinical groups. In addition, the role of emotional functioning in explaining the functional impairments associated with ADHD (e.g., peer relations) need to be examined.

In conclusion, this study has taken one important further step in trying to provide a more refined conceptual integration of the different neuropsychological and emotional impairments associated with ADHD. The results emphasized the need to include not only executive functioning but also RT variability and emotional functioning in future theoretical models of ADHD as well as in clinical practice when identifying the major impairments in this diagnostic group.

## Key points

- For neuropsychological functioning, independent effects were seen for RT variability and inhibition, but not for working memory, shifting, and delay aversion.
- Regulation of both positive and negative emotions contributed to the prediction of ADHD status, over and above the influence of neuropsychological functioning.
- No gender differences were found.
- The present results emphasize the need to better understand the role of emotion regulation in ADHD, both from a theoretical and clinical standpoint.
